# Response of Atlantic salmon *Salmo salar* to temperature and dissolved oxygen extremes established using animal-borne environmental sensors

**DOI:** 10.1038/s41598-017-04806-2

**Published:** 2017-07-03

**Authors:** Kilian M. Stehfest, Chris G. Carter, Jaime D. McAllister, Jeff D. Ross, Jayson M. Semmens

**Affiliations:** Institute for Marine and Antarctic Studies, University of Tasmania, Private Bag 49, Hobart, TAS 7001 Australia

## Abstract

Understanding how aquatic species respond to extremes of DO and temperature is crucial for determining how they will be affected by climate change, which is predicted to increasingly expose them to levels beyond their optima. In this study we used novel animal-borne DO, temperature and depth sensors to determine the effect of extremes of DO and temperature on the vertical habitat use of Atlantic salmon *Salmo salar* in aquaculture cages. Salmon showed a preference for temperatures around 16.5 to 17.5 °C, however, selection of preferred temperatures was trumped by active avoidance of low DO (<35% saturation) at the bottom of the cage. In addition to low DO, salmon also avoided warm surface waters (>20.1 **°**C), which led to a considerable contraction in the available vertical habitat. Despite their avoidance behavior, fish spent a large amount of time in waters with suboptimal DO (<60% saturation). These results show that vertical habitat contraction could likely be a significant consequence of climate change if the reduction in DO outpaces the increase in hypoxia tolerance through local adaptation. They furthermore highlight that site-specific environmental conditions and stock-specific tolerance thresholds may need to be considered when determining stocking densities.

## Introduction

Oxygen is the final receptor in the electron transport chain underlying the aerobic respiration of most organisms^1^. Hence, dissolved oxygen (DO) is a crucial and potentially limiting component of the metabolism of marine animals, due to the low availability and high cost of extraction of oxygen in aquatic environments^2, 3^. Temperature is the most important environmental factor governing the metabolic rate in ectotherms^4^ and the effects of DO and temperature on marine fish are intrinsically linked as higher temperatures increase the metabolic rate and consequently oxygen demand but also lower the solubility of oxygen, reducing supply^5^. Together, these two environmental variables have a profound effect on the ecology of marine species, from the growth and health of individuals^6^ to the spatial distribution of populations and species^5^.

Understanding how marine fish respond to extremes of DO and temperature, both in physiology and behavior, is crucial for predicting how they will be affected by climate change, which is likely to increasingly expose them to levels beyond their optimal range^7^, reducing the productivity of both wild and aquaculture populations^8^. Salmonids are one of the families of species of particular concern, due to their commercial importance both as wild caught and aquaculture species^6^ and their suspected vulnerability to environmental change (see Jonsson and Jonsson^9^ for review), with increased mortality rates in some populations already attributed to warming temperatures^10^.

A plethora of laboratory studies have tested the effects of different temperatures and DO levels on the physiology of salmonids with the aim of establishing optimal ranges and tolerance to extremes (e.g. Anttila, *et al*.^11^, Remen, *et al*.^12^, Remen, *et al*.^13^, Kutty and Saunders^14^, Javaid and Anderson^15^, Barnes, *et al*.^16^). However, the use of those studies in predicting the behavioral response to extremes of DO and temperature in the wild is limited, as the range of responses available to fish and complex interactions of competing environmental influences cannot be replicated in the laboratory^1^. Particularly the movement behavioral response of salmonids to environmental extremes is difficult to replicate in space-limited laboratory settings, however, understanding it is crucial, as any possible avoidance behavior is likely to alter other important ecological variables such as predation risk and feeding competition^1^.

Studies of the response of salmonids to extreme DO and temperature values in the field have been carried out using echosounders to determine swimming depth of salmon in aquaculture cages in concurrence with high resolution environmental sensors^17–19^. However, these studies can only measure group level rather than individual responses and only over relatively short timespans (days to weeks) and have not been able to resolve the question of whether salmon actively avoid extreme DO and temperature levels in the field^20^. Biotelemetry methods on the other hand, which deploy environmental sensors on animals, allow the collection of individual level data over longer time spans (months). While more intrusive than echosounders, biotelemetry tags have been used successfully to measure DO^21^ and temperature values^22^ experienced by free-swimming salmonids.

The aim of this study was to use newly developed acoustic telemetry tags which record swimming depth and environmental DO and temperature experienced by Atlantic salmon *Salmo salar* in aquaculture cages in Macquarie Harbour, Tasmania, Australia to determine the behavioral response of salmon to seasonal extremes of DO and temperature during the hottest summer on record in Tasmania^23^ and identify the trade-offs between the opposing influences of the two environmental parameters. Macquarie Harbour presents an ideal site for this study as it is highly stratified with a high DO, high temperature top layer and low DO, low temperature bottom layer^24^.

## Results

### Data overview

**Figure 1:**
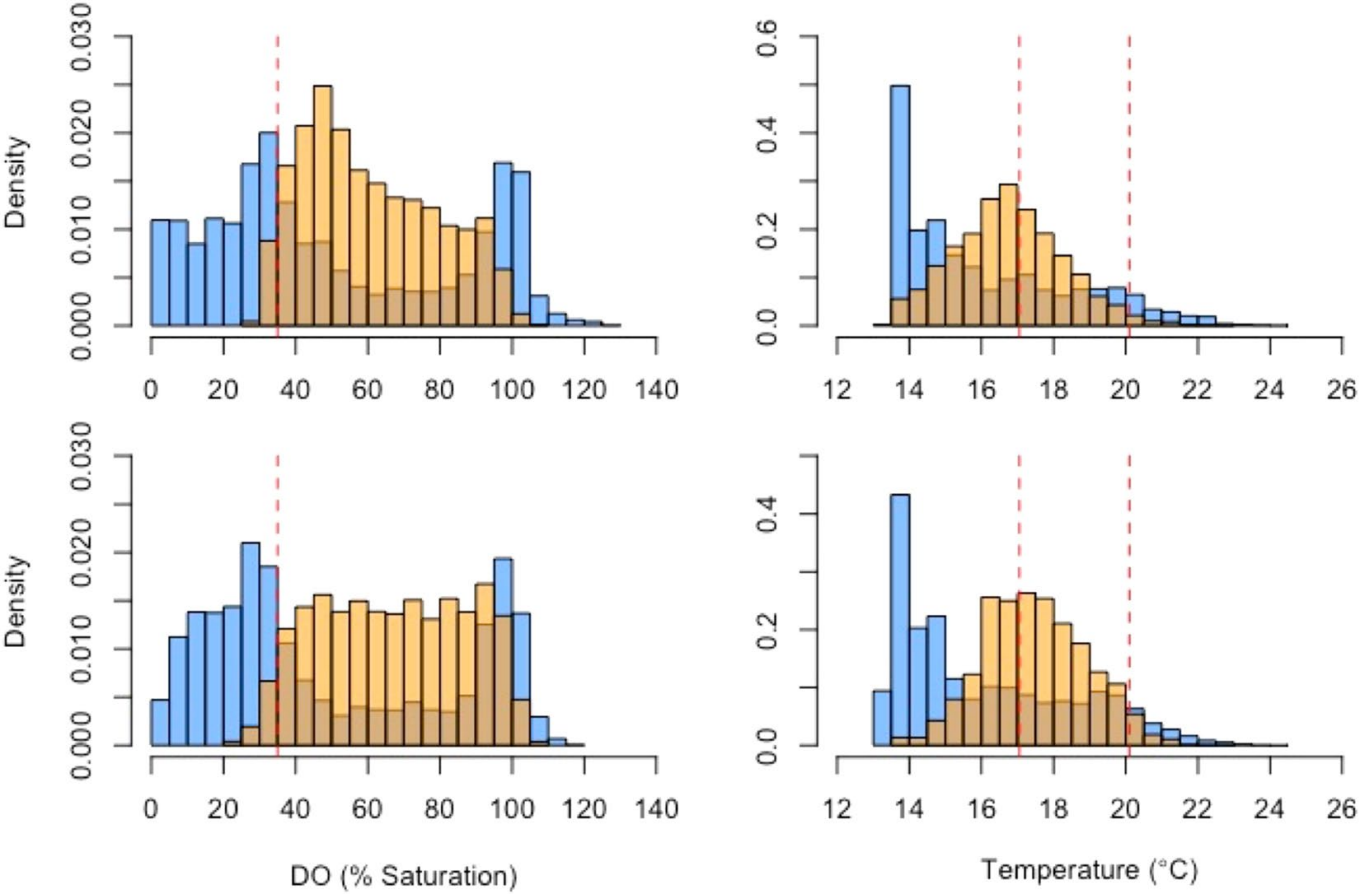
Distribution of DO and temperature values experienced by the fish and measured inside the cages. Values measured by fish are displayed in orange, those measured throughout the cage water column in blue. Data from cage 1 is shown in the top panel, data from cage 2 is shown in the bottom panel. Vertical dashed lines indicate the 35% DO saturation threshold in the DO distributions and the mean and 97.5 centile in the temperature distributions. To account for the fact that some stationary string tags malfunctioned during parts of the study period, water column data were grouped into 2.5 m depth bins and each data point weighted by the inverse of the total number of observations for its depth bin for the computation of density distributions.

Initial processing resulted in the removal of 7 tagged fish, 6 mortalities and 1 fish that lost its tag, with the majority of mortalities occurring in cage 2. All 6 mortalities occurred within 48 hours of surgery, suggesting that handling stress rather than longer-term effects of the tag attachments were the cause (Table 1). A further 5 animals died during the study, however, detection series were deemed sufficiently long to be included in analyses. While the remaining 20 fish represent only a small fraction of the total stock in each pen, previous studies have shown that individual response to environmental factors was well correlated with average group behavior of farmed salmon both in the short and long term^20^. Mean DO and temperature was similar in the two cages and in both cages mean DO and temperature values experienced by the fish were higher than those measured throughout the cage (Table 2). Fish in cage 1 swam on average approximately 1 m deeper than fish in cage 2 and consequently experienced cooler mean temperatures and lower DO values than fish in cage 2. In both cages fish spent a considerable amount of time in waters with DO values between 35 and 60% (Fig. 1) and particularly in cage 2 the distribution of DO values experienced by the fish resembled a uniform distribution with a lower bound of 30 to 40% and a higher bound of 95 to 100% whereas the temperatures experienced by the fish were approximately normally distributed, centered around a mean of 16.5 to 17.5 °C (Fig. 1). Maximum temperatures experienced by the fish (23.0 °C) were lower than those measured throughout the cages (24.2 °C).

**Table 1:**
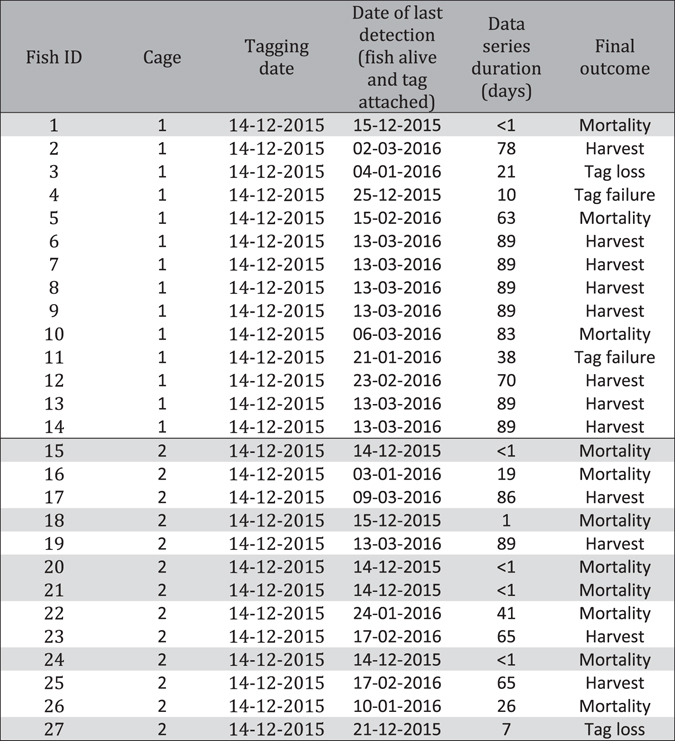
Overview of individual detection series duration.

Fish removed from analysis are highlighted in grey. Final outcomes other than harvest were established based on the animals’ depth record. Tags abruptly rising to the surface and remaining there were classed as lost tags, tags sinking to the bottom of the cage and remaining there were classed as mortalities, tags abruptly ending transmissions were classed as tag failures.

**Table 2:** Data overview.

	Cage 1	Cage 2
Number of fish	13	7
Mean swimming depth (m)	5.99 (2.30)	4.90 (1.94)
Mean DO experienced by fish (% sat)	60.80 (18.51)	67.12 (20.34)
Mean DO measured in cage (% sat)	50.58 (34.02)	49.98 (33.86)
Mean temperature experienced by fish (°C)	16.80 (1.51)	17.54 (1.43)
Mean temperature measured in cage (°C)	16.16 (2.38)	16.12 (2.41)

Standard deviations shown in brackets. To account for the fact that some stationary string tags malfunctioned during parts of the study period, cage water column data were grouped into 2.5 m depth bins and each data point weighted by the inverse of the total number of observations for its depth bin for the calculation of means and standard deviations.

Values measured by fish are displayed in orange, those measured throughout the cage water column in blue. Data from cage 1 is shown in the top panel, data from cage 2 is shown in the bottom panel. Vertical dashed lines indicate the 35% DO saturation threshold in the DO distributions and the mean and 97.5 centile in the temperature distributions. To account for the fact that some stationary string tags malfunctioned during parts of the study period, water column data were grouped into 2.5 m depth bins and each data point weighted by the inverse of the total number of observations for its depth bin for the computation of density distributions.

### Seasonal patterns

**Figure 2:**
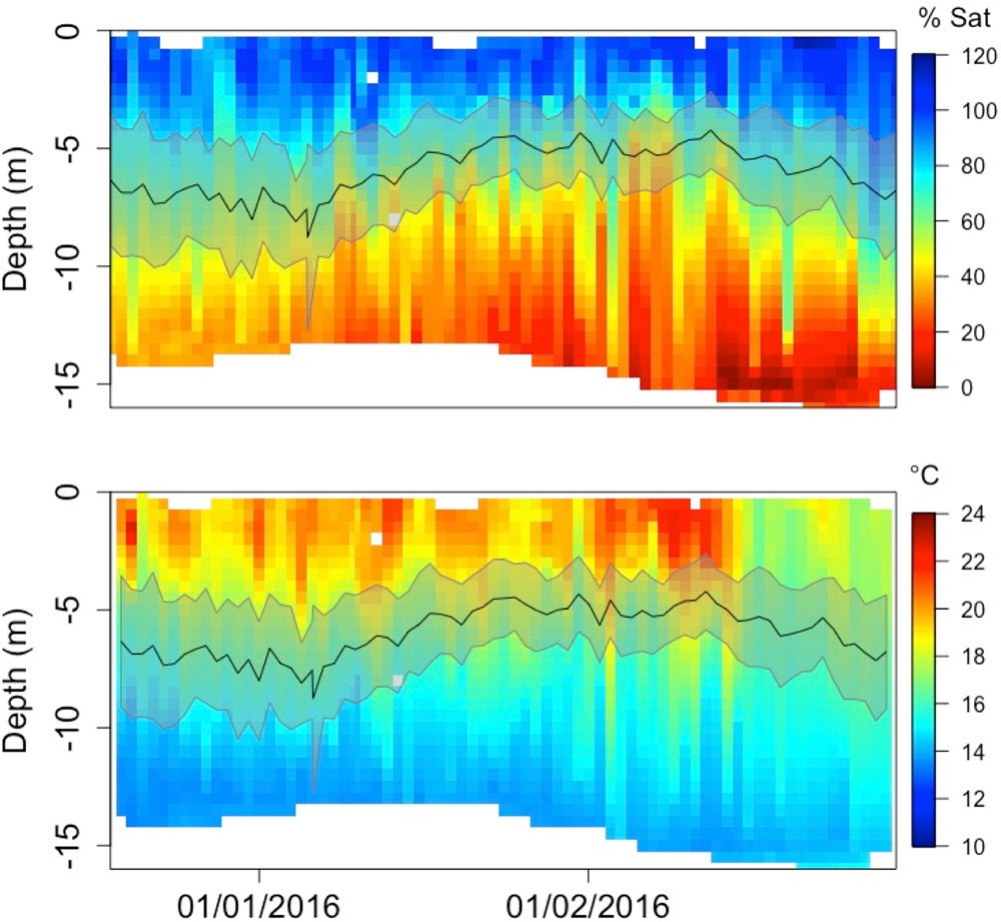
Profile of DO and temperature in experimental cage 1 during the study period. Black line indicates mean swimming depth averaged over all tagged fish in cage 1. Grey area shows ± 1 SD.

**Figure 3:**
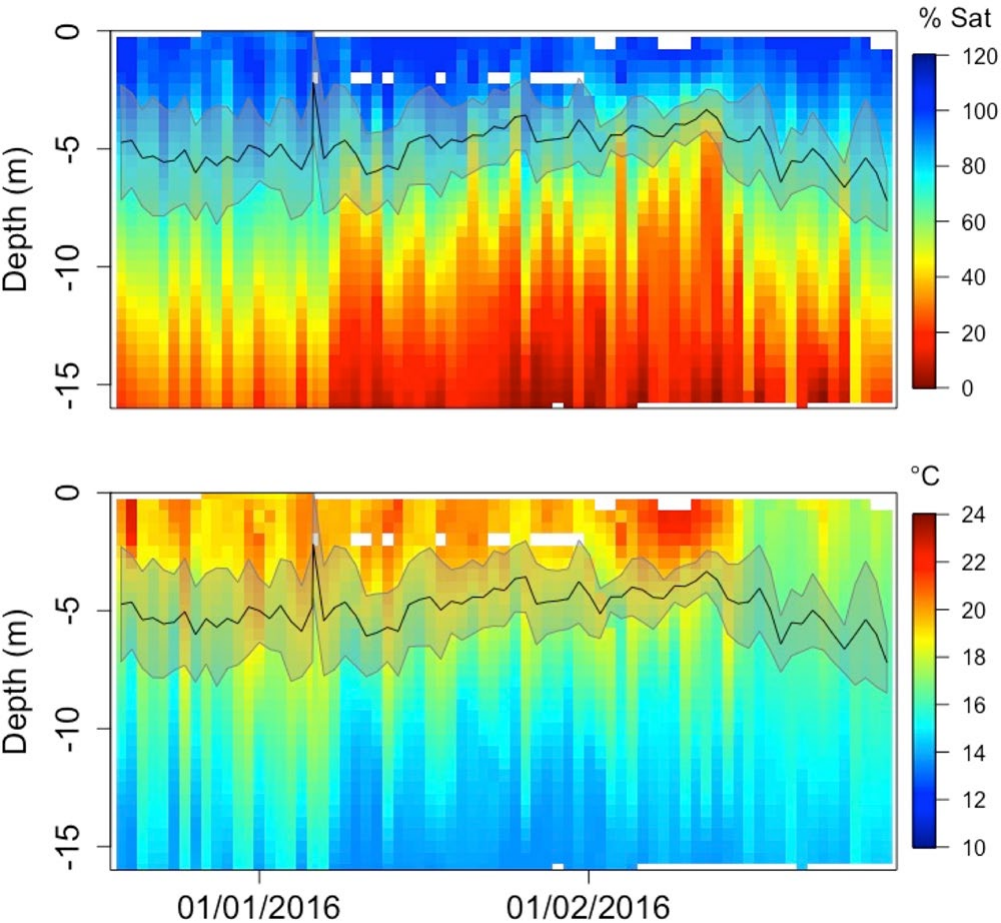
Profile of DO and temperature in experimental cage 2 during the study period. Black line indicates mean swimming depth averaged over all tagged fish in cage 2. Grey area shows ± 1 SD.

Over the course of the study period, DO and temperature underwent strong temporal changes, with DO values in the 5–10 m depth range decreasing, surface water temperatures increasing and warm surface water temperatures penetrating deeper into the water column as summer progressed (Figs 2 and 3). Fish also showed a temporal pattern in their swimming depth, with both mean depth and standard deviation decreasing over the course of summer, reaching a minimum during the peak of summer (end of January/beginning of February) and increasing again towards the end of summer. Seasonal changes in swimming depth were more pronounced in cage 1 (Fig. 2) than cage 2 (Fig. 3), which showed an overall shallower mean swimming depth. However, the seasonal changes in standard deviation were consistent across the two cages.

### Swimming behavior models

**Figure 4:**
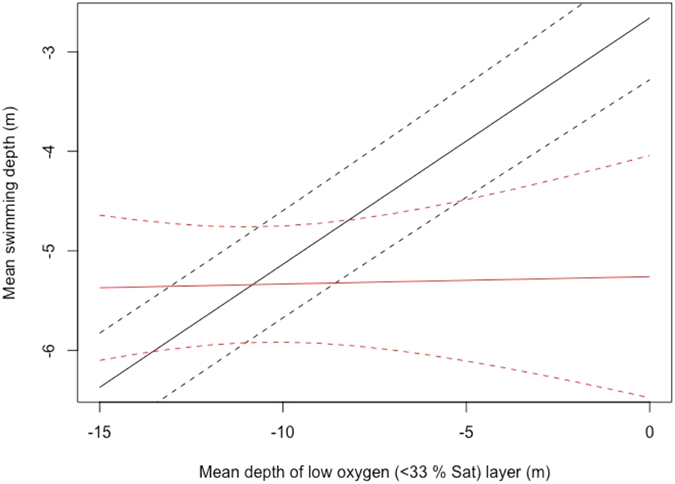
Model prediction of fish response to changes in the minimum depth of the low DO bottom layer mediated by the maximum depth of the high temperature surface layer. Black line indicates response to low DO layer depth when the high temperature surface layer was above 1 m depth, red line indicates response when the high temperature surface layer extended below 3 m. Dashed lines show 95% confidence interval.

The linear mixed models of salmon vertical movement behaviour tested two opposing hypothesis: The preference hypothesis which stipulates that salmon position themselves at or near their optimum temperature, assumed to be 17 °C and the avoidance hypothesis which stipulates that salmon position themselves to avoid both harmfully low DO (<35%) and excessive surface temperatures (>20.1 °C). The preference model results showed that the observed changes in 6 hourly swimming depths could not be successfully explained by the preference hypothesis, with the depth of the 17 °C layer having no significant effect on fish swimming depth (Table 3). The avoidance model on the other hand, showed that depth of the 35% DO layer had a significant effect on fish swimming depth, which was mediated by the effect of the maximum depth of the 20.1 °C surface layer, as indicated by the significance of the interaction term (Table 3). This significance of the interaction term in the model was predominantly driven by a significantly different response to the 35% DO level when the warm surface layer extended below 3 m (Table 3). When the warm surface layer did not extend below 3 m depth, fish moved up in the water column when the depth of the 35% DO threshold decreased. When the warm surface layer extended below 3 m, however, the response of the fish was depressed and the change in swimming depth was minimal (Fig. 4). The variance of the random intercept, which accounts for individual variation between fish was 1.417.

**Table 3:** Model parameters for the preference and avoidance models.

	Parameter estimate	t-value	p
Preference model			
Intercept	−5.672 (0.255)	−22.278	<0.001
Depth of preferred temperature layer	−0.033 (0.018)	−1.884	0.06
Avoidance model			
Intercept	−2.659 (0.311)	−8.560	<0.001
Depth of low DO threshold	0.248 (0.014)	18.245	<0.001
Depth of high temperature threshold (1–2 m)	−0.101 (0.264)	−0.386	0.700 (0.649)
Depth of high temperature threshold (2–3 m)	0.073 (0.266)	0.276	0.783 (0.649)
Depth of high temperature threshold (>3 m)	−2.601 (0.566)	−4.593	<0.001 (0.649)
Depth of high temperature threshold (1–2 m) *depth of low DO threshold	−0.003 (0.025)	−0.119	0.905 (<0.001)
Depth of high temperature threshold (2–3 m) *depth of low DO threshold	0.005 (0.025)	0.204	0.838 (<0.001)
Depth of high temperature threshold (>3 m) *depth of low DO threshold	−0.241 (0.052)	−4.613	<0.001 (<0.001)

Standard error of parameter estimates given in brackets. Overall p-value of categorical variable given in brackets behind level specific p-values. For both models the inclusion of a temporal autocorrelation structure increased the AIC by more than 2, hence the final model was fitted without the correlation structure.

## Discussion

In this deployment of novel salmon-borne DO, temperature and depth sensors we showed for the first time that salmon in aquaculture cages adjusted their vertical positioning to actively avoid both low DO levels in the bottom layer and extreme surface temperatures. Tagged fish showed a gradual upward movement and reduction in depth range in response to rising low-DO waters which was suppressed by warm surface waters penetrating deeper into the water column at the peak of summer, resulting in the concentration of the salmon stock in the 4–6 m midlayer. Our results suggest that this avoidance behavior overrides the selection of optimal temperatures in the water column.

While the tagged fish only represent a small proportion of the total stock in the two experimental cages and an effect of the tags on salmon behavior and fitness cannot be discounted as the elevated mortality of tagged fish indicates, the fact that the observed patterns were consistent across cages and fish, both those that died during the study and those harvested at the end makes it likely that they are representative of the broader stock. Additionally, the observed concentration of fish in a small band in the midlayer of the cage is consistent with the results from a short term study on salmon swimming depth in Macquarie Harbour at the peak of summer using non-invasive echosounders^19^.

It is well established that like most aquatic animals, fish have the sensory capacity to detect and avoid waters with low oxygen levels^25^ with a number of species exhibiting horizontal^26^ or vertical avoidance behavior^27, 28^ even at the larval stage^29^. Electronic tagging studies on wild salmon have provided circumstantial evidence that both Chinook *Oncorhynchus tshawytscha* ^30^ and Atlantic salmon^21^ may alter their migratory behavior to avoid waters with DO lower than 55% saturation. More recent studies on the vertical distribution of Atlantic salmon in aquaculture cages on the other hand have been inconclusive, with one study indicating avoidance of waters at or below 60%^17^ and two others finding no evidence of avoidance behavior in salmon with regards to low DO levels^18, 31^. Johansson, *et al*.^18^ concluded that the salmons’ preference for their optimum temperature overrides their response to potentially harmfully low DO levels, which was confirmed by a later experimental study, which showed an avoidance response of salmon to short-term drops in DO to intermediate levels (~60% saturation) but suggested that this was restricted to environments where more suitable DO levels are available within the animals ‘ preferred temperature band^32^. However, fish in neither of these studies experienced DO levels below 57–60%, which is higher than the lowest levels observed in our study (21–110% saturation) and the study on wild salmon^21^.

Our study showed that avoidance of DO levels below 35% was a better predictor of salmon swimming depth than preference for optimal temperatures, although, the two models we tested are not necessarily mutually exclusive and the salmon in our study may switch to the temperature preference behavior observed by Johansson, *et al*.^18^ during times when DO levels are above 60% throughout the cage. Even though we did not quantify a specific DO threshold that triggered avoidance behavior in this study, it is clear from our results that the threshold falls below what is reported as the onset of harmful conditions in the literature. Salmon are generally considered to be a hypoxia sensitive species with high oxygen requirements to meet high routine metabolic demands^16^ and comparative studies have shown that Atlantic salmon have higher critical oxygen levels for swimming performance than rainbow trout *Salmo gairdneri*, goldfish *Carassius auratus* and *Tilapia* ^14^. Moreover, growth of Atlantic salmon has been reported to be reduced below a threshold of 70%, hypoxia stress sets in below 60% as indicated by increases in plasma lactate levels^13^, immune response is depressed at or below 52%^33^ and 39% has been suggested as the critical oxygen saturation^34^ below which salmon are no longer able to maintain oxygen uptake rates through increased gill ventilation and have to rely on anaerobic ATP production^13^.

While we were not able to assess growth in the tagged fish, the fact that they experienced and survived in DO values below these thresholds suggests that they may be able to cope with lower DO levels either due to acclimation or genetic adaptation. Remen, *et al*.^12^ found no effect of acclimation on hypoxia tolerance. However, the periodic drops in DO simulated in their study were different to the chronic, gradual decrease in DO experienced by the salmon in Macquarie Harbor and a separate study showed that while the effect of hypoxia on the metabolic rate persisted, the initial stress response was down-regulated, suggesting that fish might become desensitized over time^13^. Similar to our study, Barnes, *et al*.^16^ found a higher degree of hypoxia tolerance in the Tasmanian aquaculture population in a laboratory study in freshwater and suggested as one possible explanation that a preconditioning event might have occurred since experimental fish had been exposed to low DO levels prior to procurement for the experiment. The authors also mention that the Tasmanian stock might have undergone genetic mutation^35^ as another potential reason for its increased hypoxia tolerance. This hypothesis is supported by a study of the variation in temperature and hypoxia tolerance among families of Atlantic salmon, which indicated substantial genetic variation in these traits^11^. This genetic variation could potentially allow for selective breeding in aquaculture or adaptation in the wild to respond to global warming^11^.

Deutsch, *et al*.^7^ modeled the changes in metabolically viable habitat, where environmental oxygen supply exceeds the resting metabolic demand and predicted that marine species will have to adapt to a steep overall decline and vertical contraction in suitable habitat on a global scale. On a local scale, our study shows the effects temperature and DO extremes can have on salmon in aquaculture with low bottom DO and high surface temperatures severely limiting the suitable vertical habitat. During the peak of summer, a layer of approximately only 2 m of the 17 m cage depth was available to the fish. This means that schooling densities might have been up to 8.5 times higher than theoretical stocking densities calculated based on the full cage volume. This could potentially lead to overcrowding and increased stress, which can result in heightened susceptibility to disease and suppressed cytokine expression^20^. Hence, based on a review of the environmental drivers of salmon behavior in aquaculture cages, Oppedal, *et al*.^20^ suggested site-specific stocking densities guided by prevailing environmental conditions.

## Conclusions

The results of the deployment of animal-borne, combined DO, temperature and depth sensors on Atlantic salmon in aquaculture showed that salmon actively avoided both low DO levels near the bottom of the aquaculture cage and warm surface water temperatures. This led to a considerable contraction in the vertical habitat available to them and potential overcrowding, with expected implications for fish welfare. Despite the avoidance behavior, fish spent a large amount of time in waters with suboptimal DO levels, suggesting that the hypoxia tolerance in the Tasmanian population might be higher than that reported in the literature for other stocks. These results indicate that site-specific environmental conditions need to be taken into account when determining stocking densities and that vertical habitat contraction could likely play a significant part in the effects of climate change on salmon populations.

## Methods

### Study site and stock

**Figure 5:**
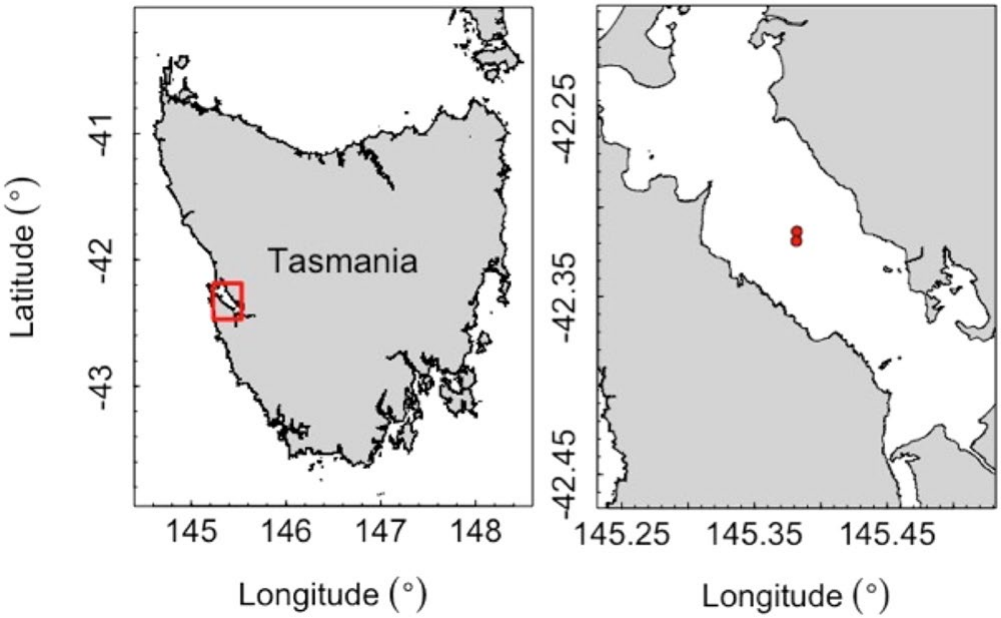
Map of experimental salmon cage locations in Macquarie Harbour on the west coast of Tasmania. Red rectangle in full-scale map of Tasmania on the left indicates extent of detailed map of Macquarie Harbour on the right. Red dots indicate experimental cage locations. Map was created in R version 3.3.1^44^, using the ‘PBSmapping’ package version 2.69.76^48^.

Macquarie Harbour is a large (~280 km^2^) estuarine inlet on the west coast of Tasmania, Australia (Fig. 5). Depths range from 5–20 m in most parts of the harbour with the exception of a deeper basin at the center, which reaches depths of up to 50 m^24^. Exchange of water with the Southern Ocean is limited to a shallow and narrow entrance at the North-western end of the harbour, freshwater input is from two main sources, the Gordon and King rivers which both carry high concentrations of dissolved organic matter^24^. Due to limited vertical mixing, waters in the harbour are highly stratified with a persistent, brackish top layer, a middle layer with low DO and intermediate salinity and a bottom layer of seawater. While the limited water exchange and vertical mixing and high load of organic matter makes the harbour a DO depleted environment, with seasonal fluctuations causing challenging conditions for salmon growth, the brackish surface layer has made the harbour attractive for salmon farming as it limits the impact of amoebic gill disease on salmon stocks, which is a problem in other parts of the state^36, 37^.

Salmonid farming in the harbour started in the 1980s and has greatly increased since then with a major expansion in 2012 and farm leases now cover a total area of 9.26 km^2^ ^38^. Whilst both ocean rainbow trout *Oncorhynchus mykiss* and Atlantic salmon are farmed the majority of stock is Atlantic salmon. Tasmanian Atlantic salmon stocks originated from the Phillip River, Nova Scotia, Canada from where they were first imported to a hatchery on the Australian mainland in the 1960s^39^. In the early 1980s the first fertilized eggs were then brought to Tasmania and the first fish transferred into ocean cages in 1985. Since the first import of ova to Australia in the 1960s new alleles have been observed in the Australian stock, suggesting the occurrence of genetic mutations^35^ and possible adaptation to local environmental conditions^16^.

### Study cages

The study was carried out between the 17^th^ of December 2015 and the 29^th^ of February 2016 in two cages owned by Huon Aquaculture, approximately 600 m apart, located in the central harbour (Fig. 5). Total depth at the two sites was between 40 and 45 m, cage depth was approximately 17 m, cage circumference was 160 m. Both cages were stocked with triploid Atlantic salmon hatched at Huon’s Millybrook Hatchery and moved into the sea cages in Macquarie Harbour in July/August the previous year (2014) and scheduled for harvest at the end of the study period (March 2016). Total number of fish in cage 1 was 36512 with an average weight at time of tagging of 3625 g, total number of fish in cage 2 was 34270 with an average weight at time of tagging of 4175 g.

### Tag deployment

To determine the behavioral response of salmon to changes in environmental conditions we deployed VEMCO acoustic telemetered sensors (17.5 × 106.5 mm; 19.78 g weight in seawater; ~160 db power output, 69 kHz frequency; VEMCO, Nova Scotia, Canada), which measure pressure (0–102 m), temperature (0–25 **°**C) and dissolved oxygen (0–140%) approximately simultaneously (within 250 milliseconds of one another) and transmit the resulting data together with the tags’ ID coded as a series of acoustic pings (one ping series per environmental variable) which were recorded together with the date and time of transmission by an acoustic receiver deployed at the center, bottom of the cages, pointing up. To reduce the probability of signals from different tags colliding, transmission intervals were randomized within a set range. The DO sensor integrated in the tag was the optical RedEye oxygen sensor patch (Ocean Optics,, Dunedin, FL, USA) which has an accuracy range of 0.01 to 0.1% and a response time of less than 20 seconds. Since the tags did not measure salinity, DO was transmitted as % saturation rather than mg/L.

Tags were deployed both as strings of stationary sensors, attached at fixed depths to a rope at the center of the cages from the surface to the bottom and on fish. Stationary tags were deployed at 1, 3, 5, 7, 9, 12 and 15 m with one additional tag deployed below the cages at 20 m. These tags had a transmission delay of 800–1000 seconds, which means that measurements of the three variables were taken every 40–50 minutes.

Fish tags were deployed on 13 and 14 fish in the two cages and had a transmission delay of 180–260 seconds, which means measurements were taken every 9–13 minutes. This represents only a small fraction of the total stock in the cages, due to cost and receiver bandwidth constraints, however, Johansson, *et al*.^40^ found that both short and long-term average individual behavior measured using data storage tags correlated well with overall group behavior. Tags were attached to the salmon using the method developed by Lacroix^22^, whereby the tag is secured to the fish via two monofilament tethers which form a lateral loop through the fish, directly underneath the pterygiophores. To ensure the tag was hovering just above the fish, it was made slightly positively buoyant by attaching a small cylindrical float to the end of it, replicating the hydrodynamics of a pop-up satellite archival tag, for which the method was designed^22^. Fish were brought to the surface using a crowd net and subsequently collected from the cage by farm staff using dip nets and immediately placed in an anesthetic bath containing Aqui-S (active ingredient Isoeugenol, Aqui-S, New Zealand). Once fish were sedate, they were moved to a padded cradle for tagging. During the tagging procedure, seawater containing a low dose of Aqui-S was continuously pumped over their gills to facilitate breathing and maintain anesthesia. Upon completion of the tagging procedure, fish were marked with Alcian Bue dye^41^ using a needleless inoculator (Dermo-jet Model G, Robbins Instruments, New Jersey, USA) for identification of tagged fish during harvest in the case of tag loss and placed in a recovery bath. Only tagged fish that fully regained balance and responded to manual agitation after recovery were released back into the cage. At the end of the study period, tags were recovered from fish during routine harvest operations and harvested tagged fish were deemed to be in good body condition (personal observation).

### Animal ethics

Prior to the field deployment, tagging procedures and tag attachment method were trialed on 6 fish kept in a tank and monitored for 3 months after tagging. Fish were monitored and fed daily and physical condition assessed by the University veterinarian every two weeks. No difference in swimming or feeding behavior was observed between tagged fish and 6 control fish kept in the same tank. After a final physical and histological assessment by the veterinarian at the end of the trial, all methods were approved by the University of Tasmania animal ethics committee (Approval No. A0014977) and all procedures during both tagging trial and field deployment were performed in accordance with relevant guidelines and regulations.

### Data processing

Prior to analyses, any data that might contain abnormal behavior was removed by excluding any fish that were detected and alive for less than 7 full days after tagging. Additionally, the first 24 hours after tagging were removed to exclude any potential effects of tagging and handling stress^42^ and for fish that were identified as mortalities, the last 24 hours before the time of death (the point where the depth measured by the tag remained constant at approximately the depth of the cage) were removed.

Following data cleanup, DO and temperature profiles were interpolated for the two cages from both fish and sensor string data on a 6 hours by 0.5 m grid using linear bivariate interpolation for irregularly distributed data, developed by Akima^43^ and implemented in R^44^ in the ‘akima’ package^45^.

### Data analysis

To determine the effect of DO and temperature on fish behavior we formulated two linear mixed models representing two contrasting hypotheses. The first model, termed the ‘preference model’ hypothesized that fish position themselves in the water column based on their preferred temperature^18^. The preferred temperature was hereby defined as the mean of all temperature values measured by the fish tags (17.05 °C; Fig. 1), which corresponds well with laboratory studies on the temperature preferences of the species^15, 18^. Consequently, the model used the 6 hourly depth of the 17.05 °C temperature layer derived from the interpolated temperature profiles as the fixed effect, fish ID as the random effect and 6 hourly mean swimming depth of individual fish as the dependent variable. Cage ID was included as a fixed effect in the original model but was not significant (α = 0.05) and therefore removed from the final version of the model. Mean swimming depth rather than raw values were used as the dependent variable to match the resolution of temperature and DO profiles and reduce temporal autocorrelation in the data.

The second model, termed the ‘avoidance model’ hypothesized that salmon position themselves vertically to avoid extremes of low DO and high temperatures. The low DO threshold was estimated to be at 35% saturation based on a previous study on the hypoxia tolerance of the Tasmanian salmon stock^16^, a value that corresponded well with the distribution of DO values experienced by the tagged salmon (Fig. 1). The high temperature threshold was estimated directly from the distribution of temperature values measured by the fish tags. Since measured temperatures were approximately normally distributed, the upper end of the 95% reference range (97.5 centile; 20.1 **°**C) was used. Analogous to the ‘preference model’, depth of the DO threshold and temperature threshold layers as well as their interaction were used as fixed effects in the model, and fish ID used as the random effect. Since the depth of the 20.1 **°**C layer fell above the shallowest deployment depth of the tags during parts of the study period it could not be estimated for each 6 hour interval. Hence, it was converted from a continuous, numeric to a categorical variable, with depths assigned to the following bins: <1 m, 1–2 m, 2–3 m, >3 m.

As with the ‘preference model’, Cage ID was initially included as a fixed effect but removed from the final model after it was found to be insignificant.

Both models were fitted in R using the ‘nlme’ package^46^, significance of model parameters was estimated using the Wald Chi-squared test. Visual inspection of model residuals was used to ensure assumptions of homoscedasticity and normality of residuals were not violated. To test whether using 6 hourly averages of swimming depth was sufficient to eliminate temporal autocorrelation in the data, we fitted both models with and without a continuous, first order, autoregressive correlation structure and compared the resulting models using Akaike’s Information Criterion (AIC) as suggested in Zuur, *et al*.^47^.
